# In Vivo Pharmacokinetic Study of *Polygonatum cyrtonema* Polysaccharide DPC1 after Oral and Intraperitoneal Administration

**DOI:** 10.3390/ph17030343

**Published:** 2024-03-06

**Authors:** Jin Yong, Chaozheng Zhang, Yuening Cao, Shuang Tang, Fei Long, Zhixing Cao, Jun Lu, Teng Peng

**Affiliations:** College of Pharmacy, Chengdu University of TCM, Chengdu 611137, China; yongjin@stu.cdutcm.edu.cn (J.Y.); chaozhengzhang@stu.cdutcm.edu.cn (C.Z.); caoyuening@stu.cdutcm.edu.cn (Y.C.); tangshuang@stu.cdutcm.edu.cn (S.T.); longfei@cdutcm.edu.cn (F.L.); caozhixing@cdutcm.edu.cn (Z.C.)

**Keywords:** *Polygonatum cyrtonema* polysaccharide, fluorescence labeling, pharmacokinetic study

## Abstract

(1) Background: *Polygonatum cyrtonema* is a medicinal plant, and its polysaccharides are used for immunomodulation and the treatment of hyperglycemia. Investigation of the tissue distribution and pharmacokinetics of *P. cyrtonema* polysaccharide can further elucidate its pharmacological mechanisms. (2) Methods: A fluorescence-labeling approach using rhodamine B (RhB) as a fluorescent molecular probe was used for the quantitative assessment of the polysaccharide from dried *P. cyrtonema* (DPC1) samples, and the pharmacokinetics and tissue distribution of DPC1 were evaluated in mice after intraperitoneal or oral administration. (3) Results: DPC1 was successfully labeled with RhB, showing degrees of fluorescence labeling at 0.453% and 0.568% as determined by the ultraviolet and enzyme marker methods, respectively. DPC1-RhB was rapidly absorbed into the bloodstream after oral and intraperitoneal administration. Pharmacokinetic characteristics showed that oral administration and intraperitoneal administration were consistent with the features of a two-compartment model. (4) Conclusion: After administration, DPC1-RhB was primarily distributed in the tissues of the heart, spleen, and lung, indicating that the drug has a targeted effect on these tissues. Overall, the findings provide a comprehensive reference for the in vivo distribution of DPC1, together with a foundation for further elucidation of its pharmacological mechanisms and the development and application of DPC1 formulations.

## 1. Introduction

The use of the *Polygonatum* rhizoma formulation was first recorded during the Song Dynasty in China in the Supplementary Records of Famous Physicians (Ming Yi Bie Lu) [1]. It consists of the rhizomes of *Polygonatum kingianum*, *P. sibiricum*, or *P. cyrtonema* [2]. It has been used in traditional Chinese medicine (TCM) to invigorate Qi, moisten the lung, and nourish the kidney and spleen [3]. Analysis has shown that *P. cyrtonema* contains a variety of polysaccharides, saponins, flavonoids, and other chemical components [4], with polysaccharides accounting for approximately 14% [5]. Modern pharmacological studies have reported that the polysaccharides of *P. cyrtonema* have various pharmacological activities such as hypoglycemic [6], hypolipidemic [7], anti-inflammatory [8], immune-regulating [9], antioxidant, and antiaging effects [10]. The complex structure of polysaccharides, as well as the lack of easily detectable ultraviolet (UV) absorbing groups and chromophores, has made quantitative analysis difficult [11,12]. Therefore, studies on the pharmacokinetics, tissue distribution, and mechanisms of action of polysaccharides are limited. To address this challenge and promote the in-depth study of *P. cyrtonema* polysaccharides, the establishment of qualitative and quantitative assays of polysaccharide samples in plasma and tissues is necessary.

Fluorescent labeling has the advantages of high stability, sensitivity, and simplicity, and is thus widely used for quantitative analyses of drugs and biomolecules [13]. The introduction of fluorescent groups allows differentiation between the labeled polysaccharide and endogenous molecules and thus facilitates in vivo pharmacokinetic research. Highly sensitive fluorescent molecular probes such as fluorescein isothiocyanate (FITC) [14], fluorescein diacetate (FDA) [15], cyanine dyes [16], and rhodamine [17] are used to label polysaccharides. Among them, rhodamine has greater photostability than FITC and other probes. Moreover, the maximum fluorescence excitation and emission wavelengths of rhodamine are in the visible area, reducing background fluorescence interference [18,19], while rhodamine labeling results in higher fluorescence productivity and lower pH sensitivity due to the high degree of structural modification [20,21]. The use of rhodamine can thus facilitate the investigation of the polysaccharides of *P. cyrtonema*, allowing observation of the distribution and localization of the polysaccharides, as well as analysis using multifunctional enzyme markers and imaging systems. Moreover, rhodamine is relatively inexpensive and is suitable for situations requiring the processing of multiple samples or for extended studies.

**Figure 1:**
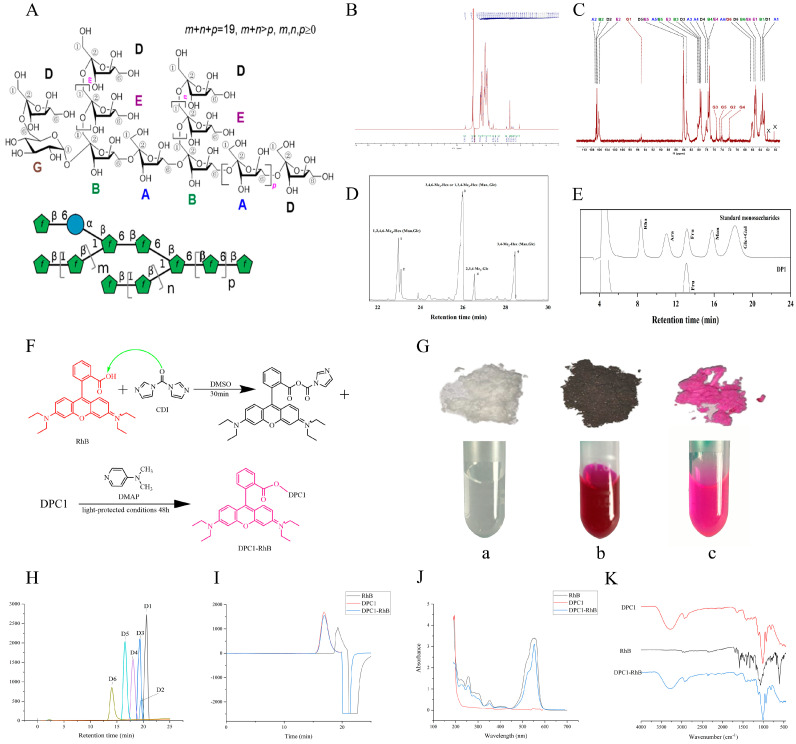
Characteristics of polysaccharide samples. (**A**) Proposed structures of the fructan DPC1 from the tonic herb *P. cyrtonema*. (**B**) The ^1^H NMR spectra of DPC1 were recorded at 600 MHz. (**C**) The ^13^C spectra of DPC1 were recorded at 298 K. (**D**) TIC spectra of DPC1. (**E**) HPLC-RID spectra of DPC1. (**F**) The labeling process of DPC1-RhB. (**G**) State diagrams of polysaccharide samples (a: DPC1, b: RhB, c: DPC1-RhB). (**H**) HPGPC graph of D-series dextrose standards. (**I**) HPGPC graph of polysaccharide samples. (**J**) UV absorption spectra of polysaccharide samples. (**K**) FT-IR spectra of polysaccharide samples.

Previous studies have isolated and extracted polysaccharide from dried *P. cyrtonema* (DPC1) [22]. Structural analyses suggest that DPC1 is a neofructan consisting of a (2→6)-linked β-D-Fru*f* residue backbone with an internal α-D-Glc*p* residue and two (2→1)-linked β-D-Fru*f* residue branches. Bioactivity tests revealed that fructan significantly enhanced the growth of *Bifidobacterium* and *Lactobacillus* strains, demonstrating prebiotic activity. The specific structure of DPC1, a homogeneous polysaccharide similar to agavin-type fructans, is shown in Figure 1A. The ^1^H NMR, ^13^C NMR, TIC, and monosaccharide composition of DPC1 are shown in Figure 1B–E. Agavin is a fructan that regulates the antioxidant response and inhibits stress-related alterations in blood metabolites (cortisol and blood lipids); it has been found to prevent oxidative damage in fish and shrimp, suggesting its potential in aquaculture [23,24,25]. Agavin shows superior fermentation capability compared with digestion-resistant maltodextrin and is an effective substrate for gut microbiota, thus promoting a healthy gut-microbial community and improving overall health [26]. In addition, agavin can also be used as a food additive to replace sucrose and fat in low-fat and low-sucrose gluten-free bakery products, which can further improve gastrointestinal health and better meet the needs of patients with celiac disease and diabetes mellitus [27]. Similarly, DPC1 also has potential in food applications. DPC1 can be purified to high levels of homogeneity, with high yields, and can be used in various applications, including the development of new drugs and biologicals. However, its tissue distribution and pharmacokinetic characteristics are not fully understood. Here, to analyze the pharmacological effects of DPC1, it was labeled with the fluorescent probe rhodamine B (RhB), and the pharmacokinetics and tissue distribution of DPC1-RhB were evaluated in mice after oral or intraperitoneal administration of three different doses. This study provides a comprehensive overview of the in vivo effects of DPC1 and serves as a reference for the detection and analytical methods for other polysaccharides.

## 2. Results

### 2.1. Characterization of DPC1-RhB

Figure 1F shows the labeling process of DPC1-RhB. As shown in Figure 1G, DPC1 was a white flocculent compound, RhB was a purple powder, and DPC1-RhB was a pink powder. All three are soluble in water, presenting a clear and transparent solution. The regression equation was calculated as *y* = 0.0447*x* + 0.1046 (R^2^ = 0.9988) and indicated a good linear relationship between the concentration of anhydrous glucose and absorbance in the range of 6.6–19.8 µg mL^−1^. The total sugar content of DPC1 was 94.38 ± 2.95% (*n* = 3). After fluorescent labeling, the sugar content was 92.55 ± 4.13% (*n* = 3), and fluorescein RhB had less effect on the total polysaccharide content of DPC1.

The HPGPC spectra of the standards with known molecular weights are shown in Figure 1H. The fitting equation, LogMw = S − 865 V + 192 V^2^ − 21.2 V^3^ + 1.16 V^4^ − 0.0253 V^5^ (R^2^ = 0.999983), was calculated. The known molecular weights were converted into logarithmic values and linearly fitted to the retention time to yield the standard curve with the equation *y* = −0.3309*x* + 9.0741 (R^2^ = 0.9985). The HPGPC spectra for DPC1, DPC1-RhB, and RhB are shown in Figure 1I. DPC1 and DPC1-RhB had a retention time of 16.8 min, whereas RhB had a retention time of 19.3 min. The HPGPC spectra revealed a single symmetrical peak for both DPC1 and DPC1-RhB with no significant differences in retention time or peak shape, indicating that the labeling reaction did not affect the molecular structure of DPC1. The outcomes of the molecular weight determination of DPC1 and DPC1-RhB are shown in Table 1. DPC1 and DPC1-RhB were found to have average molecular weights of 3185 Da and 3134 Da, respectively. This was consistent with the finding that the molecular weights of the major polysaccharide fragments of DPC1 ranged from 3000 to 4000 Da [22].

Figure 1J shows the UV absorption spectra of DPC1, DPC1-RhB, and RhB. DPC1 did not absorb in the 190–700 nm range, whereas RhB and DPC1-RhB exhibited absorption peaks at 552 nm, indicating the success of the RhB-labeling reaction.

Figure 1K shows the Fourier-transform infrared spectra of the samples. The broad and strong absorbance bands at 3385 cm^−1^ in the spectrum are attributed to the O–H stretching vibrations, whereas C–O–C glycosidic bond stretching in the sugar ring produces bands at 1129 and 1028 cm^−1^ [22,28]. The DPC1-RhB spectra show the typical peaks of the above polysaccharides. However, there were no clear characteristic peaks of RhB because its fluorescence substitution is only 0.568%, and the associated RhB signals are weak in the infrared spectra.

**Figure 2:**
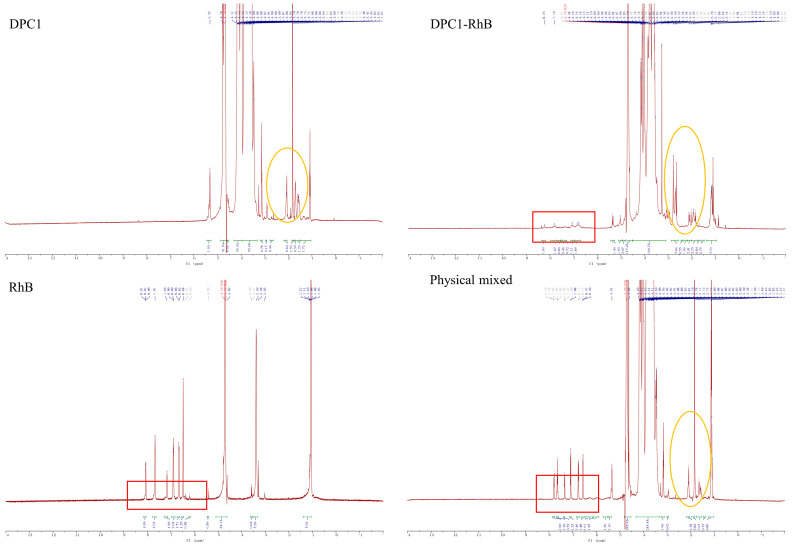
The ^1^H NMR spectra of DPC1, DPC1-RhB, RhB, and a physical mixture of polysaccharide and fluorescein (The characteristic signal of RhB were shown in the red boxes; some of the peaks that appear shift were shown in the yellow ovals).

Figure 2 displays the ^1^H NMR spectra of DPC1, DPC1-RhB, RhB, and a physical mixture of polysaccharide and fluorescein. The chemical shifts ranging from δ 1.08 to δ 5.32 ppm represent the characteristic signals of the polysaccharide DPC1, while shifts between δ 6.20 and δ 8.07 ppm signify the characteristic signals of the fluorescein RhB. The simultaneous presence of characteristic signals from DPC1 and RhB in the ^1^H NMR spectra of DPC1-RhB indicates the successful completion of the fluorescent labeling reaction. Comparison of the ^1^H NMR spectra between the DPC1-RhB and physically mixed samples indicates that the distinctive signal of RhB was more pronounced when the polysaccharide was physically mixed with RhB (highlighted by the red border in the Figure 2). The ^1^H NMR spectra of DPC1-RhB revealed shifts in some peaks of DPC1, with the peaks moving to higher ppm values. In contrast, the physically mixed sample set did not exhibit such a shift, as indicated by the yellow circle in the Figure 2. This may be due to a modification in the chemical environment of the polysaccharide in DPC1-RhB, leading to a shift in some of the peaks. Significant differences in the ^1^H NMR spectra between DPC1-RhB and physically mixed samples indicate a successful fluorescent labeling reaction, thus ruling out the possibility of physical mixing.

### 2.2. Determination of the Degree of Fluorescence Substitution

There was an excellent linear correlation between the RhB concentration and absorbance in the range of 12–28 µg mL^−1^ as shown by the regression equation *y* = 0.0285*x* − 0.0161 (R^2^ = 0.9997). The degree of fluorescence substitution of DPC1-RhB was determined to be 0.453 ± 0.029% (*n* = 3). However, RhB concentrations and fluorescence intensities in the range of 1.0–10.0 µg mL^−1^ showed a good linear correlation as shown by the regression equation *y* = 0.618*x* − 1.3267 (R^2^ = 0.9986). The degree of fluorescence substitution of DPC1-RhB was determined to be 0.568 ± 0.014% (*n* = 3).

### 2.3. Stability of DPC1-RhB In Vitro

**Figure 3:**
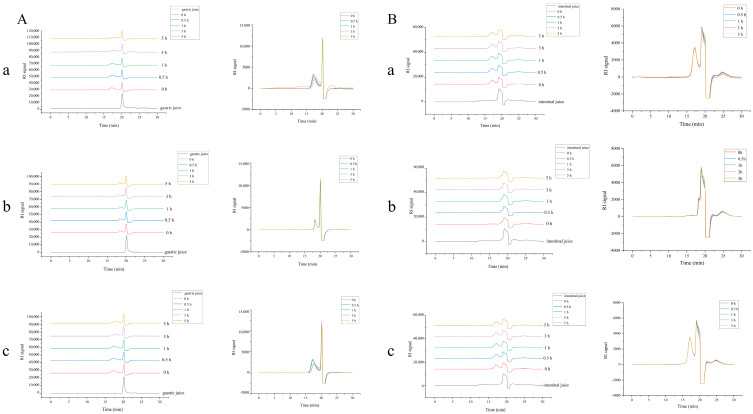
The stability of polysaccharide samples in the gastrointestinal fluids. (**A**) GPC chromatograms of samples in artificial gastric fluid over 5 h (a: DPC1, b: RhB, c: DPC1-RhB). (**B**) GPC chromatograms of samples in artificial intestinal fluid over 5 h (a: DPC1, b: RhB, c: DPC1-RhB).

In vivo stability of the polysaccharide is crucial for pharmacokinetic research using fluorescence-labeled polysaccharides. The retention time of artificial gastric fluid was 20.1 min (Figure 3A), whereas that of DPC1 and DPC1-RhB was 16.8 min (Figure 1I). Comparison of the offset stacking and overlap plots (Figure 3A(a,c)) indicated that the retention time of DPC1 and DPC1-RhB increased gradually from 16.8 to 17.8 min over a period of 5 h, and the peak heights also gradually decreased. However, the retention time and peak shape of RhB did not change significantly within 5 h (Figure 3A(b)). In addition, the retention time of the artificial intestinal fluid was 19.1 min (Figure 3B). No significant changes in the retention duration and peak shapes of DPC1, DPC1-RhB, and RhB were observed over 5 h.

### 2.4. In Vivo Imaging Studies

**Figure 4:**
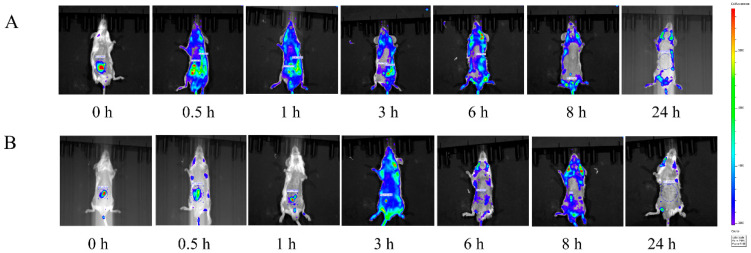
In vivo imaging results in mice. (**A**) Biodistribution of DPC1-RhB in mice after 0–24 h of intraperitoneal administration. (**B**) Biodistribution of RhB in mice after 0–24 h of intraperitoneal administration.

In this study, the visual biodistribution and absorption of DPC1-RhB in mice were explored using IVIS Spectrum CT. Figure 4A,B shows whole-body images of mice over 0–24 h after intraperitoneal injection of DPC1-RhB and RhB. The fluorescence signals were mainly concentrated in the abdomen after administration of DPC1-RhB, and the ROI value shows that the fluorescence intensity reached a maximum at 1 h and then declined over time. The fluorescence signals began to spread after 0.5 h and extended to the kidney, intestine, spleen, and other tissues in less than 1 h. Fluorescence signals were seen in the heart and lung 3 h later. After 8 h, the intensity of fluorescence signals in all tissues was markedly reduced. After 24 h, there were no discernible fluorescence signals. After administration, the fluorescent signals in the RhB group were primarily concentrated at the site of abdominal drug administration. Over time, there was no discernible trend in the drug distribution, and essentially no signal could be detected after 24 h.

### 2.5. Verification of Quantitative Analysis Method of DPC1-RhB in Plasma and Tissues

#### 2.5.1. Linear Relationship

The X-axis represents DPC1-RhB concentration in the plasma and various tissue homogenates, and the Y-axis represents the fluorescence intensity. The standard curves of DPC1-RhB in each biological sample are shown in Table 2. DPC1-RhB in each sample showed good linearity in the range of 0.5–300 µg mL^−1^ (R^2^ > 0.9932).

#### 2.5.2. Precision and Accuracy Test

The intra- and inter-day accuracy and precision of DPC1-RhB in different samples were evaluated by adding varying amounts of DPC1-RhB to blank plasma and tissue samples. The accuracy and precision of the proposed method were calculated as the relative error (RE, %) and percentage relative standard deviation (RSD, %), respectively. The accuracy, intra-day precision, and inter-day precision varied from −9.52 to 15.93%, 1.72 to 15.84%, and 1.17 to 17.52%, respectively (Table 3), which met the requirements for the analysis of biological samples.

#### 2.5.3. Stability Test

The results of the stability test are shown in Table 4. For this experiment, samples were stored at room temperature for 6 h, −4 °C for 24 h, and −20 °C for 7 d. The recovery rate of DPC1-RhB under the different storage conditions ranged from 87.11% to 113.42%, and the RSD was less than 18.56%, which satisfied the stability requirements.

### 2.6. Plasma Level of DPC1-RhB

**Figure 5:**
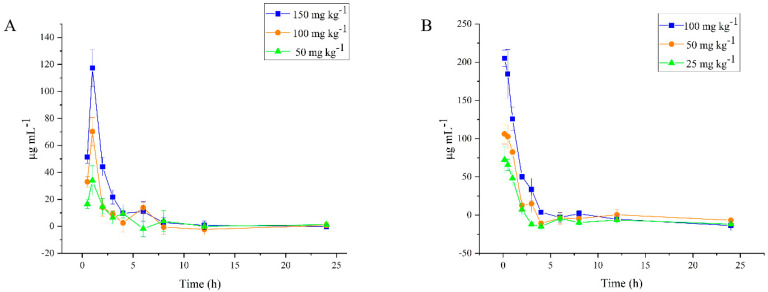
Plasma concentration time curve. (**A**) Plasma concentration-time profiles after oral administration of three different doses of DPC1-RhB in mice. (**B**) Plasma concentration-time profiles after intraperitoneal administration of three different doses of DPC1-RhB in mice.

After oral administration of 50, 100, and 150 mg kg^−1^ of DPC1-RhB, the measured plasma drug concentrations and the corresponding time points were plotted. The concentration–time profiles are shown in Figure 5A. The results showed a significant drug absorption peak after a single administration of three different concentrations of DPC1-RhB. The plasma concentration of DPC1-RhB increased steadily within 30 min after oral administration and peaked at 1 h, which was consistent with previous results [29]. The C_max_ values were 34.697, 70.236, and 117.374 mg L^−1^, respectively. Subsequently, the plasma concentration of DPC1-RhB decreased rapidly in 1–4 h and stabilized with a slow decrease over time in 6–24 h. In addition, the plasma concentration of DPC1-RhB showed a concentration dependence in the dose range of 50 to 150 mg kg^−1^. The higher the administered dose, the greater the plasma concentration of the corresponding drug.

The concentration-time profiles after intraperitoneal administration of 25, 50, and 100 mg kg^−1^ of DPC1-RhB are shown in Figure 5B. After a single intraperitoneal administration, DPC1-RhB was swiftly cleared from the plasma, experiencing the most rapid decline in blood concentration values within 2 h, followed by a gradual decrease over 3–6 h, and ultimately stabilizing. Similar to the earlier findings with oral administration, the plasma concentration of DPC1-RhB after intraperitoneal administration was proportional to the administered dose, and the concentration values at the same time point increased with the administered dose.

**Figure 6:**
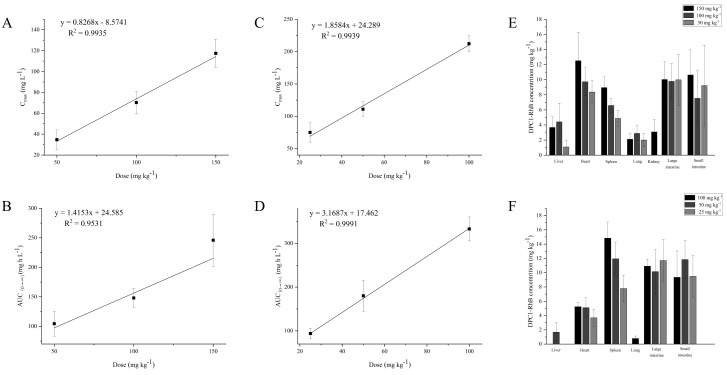
Experimental results of pharmacokinetics in mice. (**A**) Profile of C_max_ versus the three different doses after oral administration in mice. (**B**) Profile of AUC _(0→∞)_ versus the three different doses after oral administration in mice. (**C**) Profile of C_max_ versus the three different doses after intraperitoneal administration in mice. (**D**) Profile of AUC _(0→∞)_ versus the three different doses after intraperitoneal administration in mice. (**E**) Tissue distribution of DPC1-RhB after 24 h of oral administration. (**F**) Tissue distribution of DPC1-RhB after 24 h of intraperitoneal administration.

The pharmacokinetic parameters of DPC1-RhB were calculated using DAS 2.0 pharmacokinetics software (Table 5 and Table 6). After oral administration of 50, 100, and 150 mg kg^−1^ of DPC1-RhB, the drug peaked within 0.5–1 h and the elimination half-lives (T_1/2_) were relatively short, all falling below 6 h. This suggests that DPC1-RhB undergoes rapid absorption following oral administration, swiftly entering the circulatory system. The total polysaccharide concentration in the plasma was shown by the area under the plasma level-time curve (AUC_(0→∞)_) [30]. The pharmacokinetic parameters C_max_ and AUC_(0→∞)_ were utilized to generate plots for the three dose levels of DPC1-RhB. As depicted in Figure 6A,B, both C_max_ and AUC_(0→∞)_ exhibited a positive correlation with the dose for each dose group, showcasing linear relationships of *y* = 0.8268*x* − 8.5741 (R^2^ = 0.9935) and *y* = 1.4153*x* + 24.585 (R^2^ = 0.9531), respectively. After intraperitoneal administration of 25, 50, and 100 mg kg^−1^ of DPC1-RhB, the half-life was shorter compared to that observed with oral administration, indicating a faster absorption and metabolism of the drug following intraperitoneal administration. After a single intraperitoneal administration of DPC1-RhB at high, medium, and low doses, C_max_ and AUC _(0→∞)_ correlated positively with dose in each group, indicating significant dose-dependence, similar to oral administration. The linear relationships were *y* = 1.8584*x* + 24.289 (R^2^ = 0.9939) and *y* = 3.1687*x* + 17.462 (R^2^ = 0.9991), respectively (Figure 6C,D).

### 2.7. Tissue Distribution of DPC1-RhB

**Figure 7:**
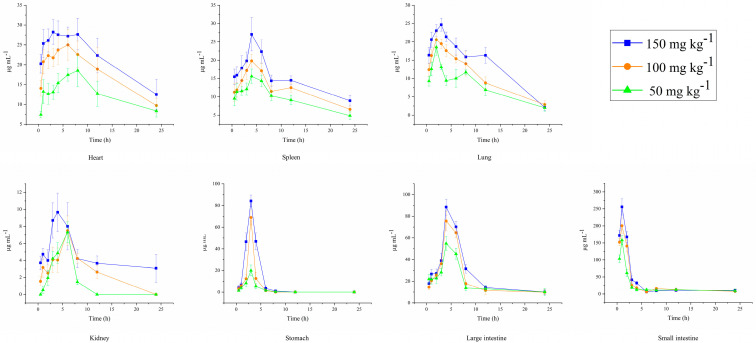
Drug concentration–time profiles in various tissues of mice after oral administration of three different doses of DPC1-RhB.

**Figure 8:**
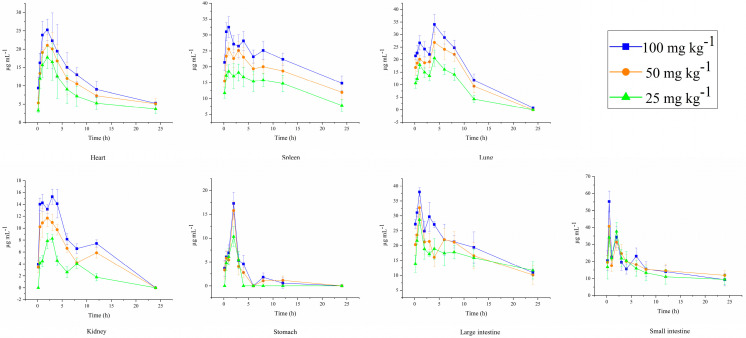
Drug concentration–time profiles in various tissues of mice after intraperitoneal administration of three different doses of DPC1-RhB.

The drug concentration–time profiles in various tissues of mice after oral administration of three different doses of DPC1-RhB are shown in Figure 7. After oral administration, DPC1-RhB initially dispersed mainly in the gastrointestinal tract, followed by absorption into the blood circulation and subsequent distribution to the liver, heart, spleen, lung, and kidney. As shown in Figure 6E, DPC1-RhB was detected in the majority of tissues 24 h after administration, in the following order: heart > intestine > spleen > liver > lung > kidney. The maximal DPC1-RhB concentration in each tissue after 24 h was 12.516 mg kg^−1^, confirming the prior in vivo imaging finding that the medication was entirely metabolized in each tissue. After oral administration, the levels of DPC1-RhB in the small intestine were higher at 30 min, peaked at 1 h, then rapidly decreased, finally dropping to low levels after 3 h. In contrast, the DPC1-RhB concentration gradually increased and peaked at 4 h in the large intestine. Additionally, DPC1-RhB exhibited a gradual increase and subsequent slow decrease in the heart, spleen, and lung over 0.5–12 h after administration. The drug concentration consistently remained at a high level, indicating that DPC1-RhB possesses superior targeting properties towards the aforementioned tissues. Combined with the pharmacokinetic parameters of DPC1-RhB after oral administration, it can be inferred that DPC1-RhB is absorbed into the bloodstream rapidly, as evidenced by its short T_1/2_ and MRT_(0→∞)_. This suggests quick absorption and widespread distribution in various tissues, particularly in the gastrointestinal tract, where it accumulates and persists for an extended duration. The drug concentration–time profiles in various tissues of mice after intraperitoneal administration of three different doses are shown in Figure 8. After intraperitoneal administration of DPC1-RhB, the distribution in the gastrointestinal tract was significantly lower compared to oral administration, and it was primarily found in the tissues of the heart, lung, and spleen. As shown in Figure 6F, DPC1-RhB was detected in the majority of tissues 24 h after intraperitoneal administration, in the following order: spleen > intestines > heart > liver > lung. Detailed data on DPC1−RhB tissue distribution in mice is available in the Supplementary materials.

### 2.8. Toxicity Studies

**Figure 9:**
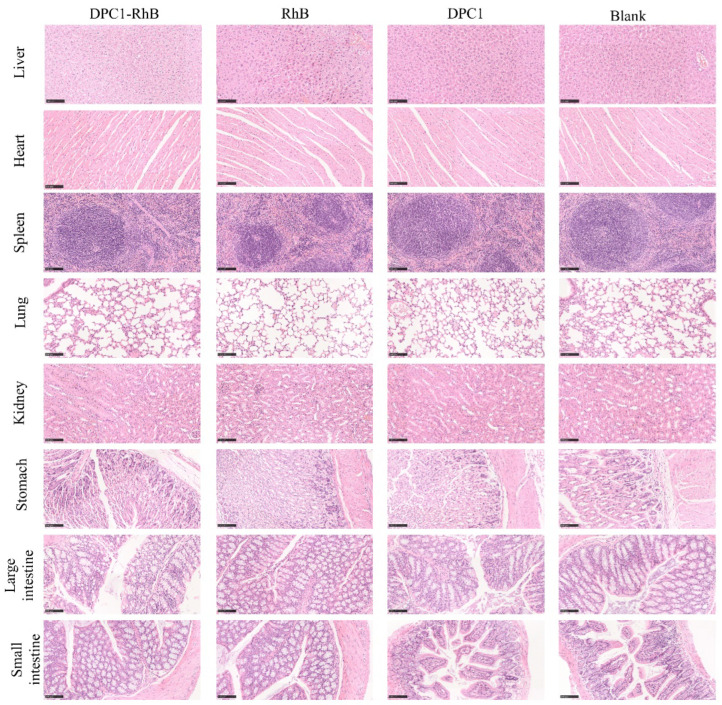
Scan of HE-stained sections of mice (The length of the scale bars was in a ratio of 1:3).

Figure 9 shows the HE-stained sections of eight organs (liver, heart, spleen, lung, kidney, stomach, large intestine, and small intestine) of mice after the intraperitoneal administration of DPC1-RhB. None of the organs in the four groups of mice showed significant lesions, inflammation, cell loss, or other abnormalities compared with organs from the blank group. These findings suggest that DPC1 and RhB are nontoxic and that labeling the polysaccharide with rhodamine does not cause detrimental changes. Therefore, it is feasible to determine DPC1 pharmacokinetics in mice by labeling DPC1 with RhB.

## 3. Discussion

The results indicate that the fluorescent labeling reaction was successful, and the fluorescent labeling substance RhB had no significant impact on the total sugar content, molecular weight, and GPC chromatogram of polysaccharide DPC1. The fluorescence substitution degree was 0.568%. The fluorescent probe not only interacts with the target molecule but should have no or only negligible effect on the target molecule that can thus be ignored. To fulfill the detection sensitivity, the degree of fluorescence substitution of DPC1-RhB should be <1%, which may guarantee a relatively low labeling rate. At the same time, it could reduce the effect of fluorescence labeling on the polysaccharide itself [31,32,33].

The retention time of DPC1 and DPC1-RhB in artificial gastric fluid increased over a 5-h period, and the peak heights decreased gradually in the in vitro simulated digestion experiment. However, the retention time and peak shape of RhB did not change significantly within 5 h. In addition, the retention times and peak shapes of DPC1, DPC1-RhB, and RhB in artificial intestinal fluid did not change significantly. This suggests that pH was an important factor [34]. This may be related to the degradation of DPC1 in an acidic environment, which eliminates a part of the sugar chain group, resulting in prolonged retention time and decreased molecular weight. The degradation of polysaccharides in acidic conditions is considered a common phenomenon [35]. Meanwhile, the peak shape of DPC1-RhB in artificial gastrointestinal fluid remained relatively stable within 5 h, indicating that fluorescein RhB binds securely to the polysaccharide DPC1 and does not undergo further decomposition.

In vivo imaging revealed that DPC1-RhB is minimally influenced by RhB after entering the mice, and the resulting tissue distribution characteristics are guided by DPC1. Meanwhile, the variability in the in vivo imaging results for DPC1-RhB and RhB mice also confirmed the stability of DPC1-RhB during the actual absorption process. However, the in vivo imaging technique is limited by the high scattering of visible light by biological tissues, and it requires a high degree of precision in controlling the timing of anesthesia and imaging. Therefore, in this study, we established a quantitative assay for DPC1-RhB in biological samples to further characterize the plasma concentration distribution and tissue distribution of the drug in mice. The method meets the requirements of precision, accuracy, and stability.

The mean plasma concentration–time profiles in mice following single oral and intraperitoneal administration of various doses of DPC1-RhB were consistent with a two-compartment pharmacokinetic model. DPC1 demonstrates linear pharmacokinetic characteristics in mice, and similar dose-dependent outcomes were observed in the pharmacokinetic studies of other polysaccharides [34,36,37]. Among them, a double peak appeared at 6 h in the medium- and high-dose groups after oral administration, and similar results were observed in these studies [34,38]. This phenomenon may be associated with the administered dose [39], gastric emptying [40], enterohepatic circulation [41], and P-gp protein expression [42]. A similar biphasic elimination phenomenon following intraperitoneal administration has been observed in the literature [29,34,43].

The distribution of DPC1-RhB in the heart, spleen, and lung tissues was more extensive in both delivery routes. The drug concentration in the heart, spleen, and lung consistently remains high over the 0–12 h period and is fully metabolized in each organ after 24 h. It is understood that mild polysaccharides may be degraded during metabolism and then enter tissues such as the spleen and lung in the form of oligosaccharides [44]. The precise targeting of DPC1 to the heart, spleen, and lung tissues also explains the remarkable therapeutic effects of *Polygonatum cyrtonema* in ‘tonifying the spleen and moistening the lung’ from a scientific perspective. Unlike the results observed with oral administration, the distribution of DPC1-RhB in kidney tissues was more prominent after intraperitoneal injection, with a tendency of increasing and then decreasing within 0.5–12 h. If DPC1 needs to target the kidney, the choice of administration should be intraperitoneal injection > oral administration. The liver can excrete substances with a molecular weight (Mw) ranging from 500 to 5000 Da [34]. However, the DPC1 concentration in the liver was consistently low irrespective of dose or mode of administration. This differs from the literature [29], which reported a significant distribution of *Polygonatum sibiricum* polysaccharide PRP in the liver tissue following oral administration. This may be due to the different nature of the two polysaccharides. PRP is a complex polysaccharide extracted from *P. sibiricum*, and its exact structure is not known. However, DPC1 is a homogeneous polysaccharide extracted from *P. cyrtonema*. Previous studies have revealed that DPC1 is a fructan primarily composed of two monosaccharides, Fru and Glc [22]. The liver is the main barrier in the absorption of xenobiotics. Studies have shown that polysaccharides containing galactose and mannose can bind to the asialoglycoprotein (ASGPR) receptor with high affinity followed by uptake into the liver via endocytosis [45,46]. DPC1 is a fructan and has a weak ability to bind to this protein receptor. Moreover, as the liver is a complex organ, hepatocytes in various regions may have varying drug-metabolizing enzyme activities [47]. These reasons may contribute to a low distribution of DPC1 in the liver.

## 4. Materials and Methods

### 4.1. Materials and Animals

*P. cyrtonema* rhizomes were collected from Yibin, Sichuan Province, China, and authenticated by Dr. Fei Long in Chengdu University of TCM, Chengdu, China. DEAE was purchased from Beijing Ruida Henghui Biotechnology Co., Ltd. (Beijing, China), RhB from Shanghai Maikun Biotechnology Co., Ltd. (Shanghai, China), and pepsin and trypsin were purchased from Solarbio (Beijing, China). Phenol, anthrone, sulfuric acid, NaCl, KH_2_PO_4_, NaOH, and DMSO were purchased from Chengdu Cologne Biotechnology Co., Ltd. and CDI was purchased from Shanghai Maikelin Biotechnology Co., Ltd. All other chemicals used were of reagent grade and were obtained commercially.

BALB/c mice (male, 7 weeks old, 20–22 g, license no: SCXK (Jing) 2019-0008) were obtained from the Hua Fu Kang Beijing Biotechnology Co., Ltd. (Beijing, China). The mice were kept in an animal room with constant temperature (20–22 °C), humidity (55–60%), and a 12 h–12 h alternating light–dark cycle for a week before the experiment. Every animal had free access to common food and water. To reduce the effect of the age and sex of the BALB/c mice on the experimental results, age- and sex-matched mice were used for this study. All animal treatments and experiments were conducted in adherence to the National Research Council’s Guide for the Care and Use of Laboratory Animals. The protocols employed in these studies were approved by the Committee of the Chengdu University of Traditional Chinese Medicine (2021-51).

### 4.2. Extraction and Purification of DPC1

Briefly, *P. cyrtonema* powder was extracted twice with ultrapure water (1:10, *w*/*v*), and the extracted filtrates were combined and concentrated. For gradient alcohol precipitation, 95% ethanol was added to the sample to a final precipitate concentration of 80%. The obtained precipitate was washed three times with anhydrous ethanol to yield the crude polysaccharide. A 10 mg mL^−1^ solution of the crude polysaccharide was applied to a DEAE-52 cellulose column for separation and purification (mobile phase: ultrapure water; flow rate: 1 mL min^−1^). The phenol–sulfuric acid method was used, and the absorbance of the eluate at 490 nm was used to construct an elution curve. Eluents at the sharp peak of the elution curve were combined. Finally, DPC1 was obtained after concentration under reduced pressure and freeze-drying.

### 4.3. Preparation of Fluorescently Labeled DPC1

Fluorescence labeling of DPC1 was performed as previously described [48]. DPC1-RhB was obtained by esterification of the active carboxyl group of RhB with the hydroxyl group of DPC1. Using CDI activation and DMAP in alkaline conditions, RhB and DPC1 were dissolved in DMSO and allowed to react at room temperature in the dark for 48 h after which the solution was dialyzed using a 1000-Da dialysis membrane against pure water. The resultant purple solution was then frozen to yield DPC1-RhB, which was subsequently stored in a dry dish away from light.

### 4.4. Characterization of DPC1 and DPC1-RhB

The anthrone–sulfuric acid method was used to determine the total sugar content. Briefly, 8 mL of 0.2% anthrone–sulfuric acid solution was added to the polysaccharide sample (0.05 mg mL^−1^, 2 mL). After thorough mixing, the sample was placed in a boiling-water bath for 10 min followed by immediate transfer to an ice-water bath for a further 10 min. The standards were solutions of D-glucose with concentrations of 6.6–19.8 µg mL^−1^. Absorbances at 588 nm were measured using distilled water as the blank. High-performance gel permeation chromatography (HPGPC) was performed using a Waters Breeze 2 (Milford, MA, USA) system with an Ultrahydrogel^TM^ column (7.8 × 300 mm) to determine the molecular weight of the polysaccharides. The weight-average molecular mass (Mw), number-average molecular mass (Mn), and molecular weight distribution were determined. The conditions and procedures for chromatography were chosen based on a previously published method [22].

To verify the fluorescence labeling reaction, the UV-visible spectra were examined to confirm the presence of RhB in DPC1-RhB. DPC1, RhB, and DPC1-RhB were dissolved in distilled water and were scanned over the 200–700 nm range with a UV spectrophotometer against water as the blank. According to the 2020 Edition of the Chinese Pharmacopoeia, Part IV of the solid press method, samples were scanned in a frequency range of 4000–400 cm^−1^ to obtain the spectra. ^1^H NMR spectroscopy analysis was conducted to gain a deeper understanding of the fluorescence labeling reaction of polysaccharides. Samples of DPC1, RhB, DPC1-RhB, and a physical mixture of polysaccharide and fluorescein (with a DPC1 to RhB ratio of 100:0.568) were analyzed using ^1^H NMR spectroscopy. An amount of 20 mg of each sample underwent deuterium exchange through freeze-drying with D_2_O (99.9%), followed by re-dissolution in D_2_O (99.9%). ^1^H NMR spectroscopy was conducted using a 600 MHz Bruker Advance spectrometer.

### 4.5. Fluorescence Labeling Rate of DPC1

The degree of fluorescence substitution is a reflection of the efficiency of fluorescence labeling. Solutions with various concentrations of RhB in distilled water were prepared. A UV–Visible spectrophotometer (UV-3100PC, Mepeda, China) and a multifunction microplate reader (SpectraMax iD5, Molecular Devices, San Jose, CA, USA) were employed to measure the fluorescence substitution degree in labeled polysaccharides, with an excitation wavelength of 552 nm and an emission wavelength of 580 nm. The corresponding absorbances or fluorescence intensities were then determined. To calculate the corresponding RhB concentration and degree of fluorescence substitution, DPC1-RhB was configured into a solution of the proper concentration, and the fluorescence intensity was measured at a wavelength of 552 nm.

### 4.6. Determination of DPC1-RhB Stability In Vitro

The in vitro stability of DPC1-RhB was assessed by simulating the digestion of polysaccharide samples in artificial gastric and intestinal fluids. Artificial gastric and intestinal fluids were prepared as described in the Chinese Pharmacopoeia (2020) [49]. The artificial gastric fluid was composed of pepsin (10 g) and dilute hydrochloric acid (16.4 mL) in 1.0 L of ultrapure water with the pH adjusted to 2.0 with 0.1 M HCl. The artificial intestinal fluid was composed of trypsin (10 g) and KH_2_PO_4_ (6.8 g) in 1.0 L of ultrapure water with the pH adjusted to 6.8 with 0.1 M NaOH.

RhB, DPC1, and DPC1-RhB solutions were added to the above fluids in 1:1 ratios. Using ultrapure water as a blank control, the samples were removed after incubation at 37 °C for 0, 0.5, 1, 3, and 5 h, respectively. The mixture was immediately placed in a boiling-water bath to stop the reaction. HPGPC was used to determine the molecular weights of the digested samples.

### 4.7. In Vivo Imaging Studies

The 2D-fluorescent imaging tomography (IVIS^®^) technique is an effective method for studying the dynamic distribution of drugs in vivo and measuring organ fluorescence emission values [50]. All experiments were performed by following published protocols with minor modifications [51]. IVIS Spectrum CT (Ex: 552 nm, Em: 580 nm, PerkinElmer, Waltham, MA, USA) was used for imaging. Male BALB/C mice were divided into two groups, DPC1-RhB and RhB, with 5 mice per group. Before the experiment, all mice were fasted for 12 h with free access to drinking water. DPC1-RhB was injected intraperitoneally at 150 mg kg^−1^, and a value of 0.568% substitution was used to determine RhB at 0.852 mg kg^−1^. All drugs were dissolved in 0.2 mL saline. The belly hair of mice was removed ahead of time and the mice were anesthetized by inhalation of isoflurane gas. Real-time optical imaging was conducted at 0, 0.5, 1, 3, 6, 8, and 24 h after intraperitoneal administration.

### 4.8. Establishment of Quantitative Analysis Methods for DPC1-RhB in Plasma and Tissues

These analyses were performed as previously described [34,52].

#### 4.8.1. Linear Relationship

The DPC1-RhB sample was weighed and diluted in phosphate-buffered saline (PBS) to prepare a 1 mg mL^−1^ stock solution. The stock solution was diluted with PBS to various concentrations (1.7, 3.4, 16.7, 33.4, 50.0, 167.0, 334.0, 500.0, 750.0, and 1000.0 µg mL^−1^). Mice in the blank group were euthanized after blood collection (0.6 mL). The liver, heart, spleen, lung, kidney, stomach, large intestine, and small intestine were collected. A tissue grinder was used to homogenize the tissues. Polysaccharide samples at concentrations of 0.5, 1.0, 5.0, 10.0, 15.0, 50.0, 100.0, 150.0, 225.0, and 300.0 µg mL^−1^ were prepared by adding 30 µL of different concentrations of DPC1-RhB solution to 70 µL of plasma and tissue homogenate. A volume of PBS equal to that of the DPC1-RhB solution was used as the blank control, and the assay was performed as described in Section 4.5. The weighted least-squares method was used for regression analysis, using the linear regression equation as the standard curve where the X-axis represented the concentration of DPC1-RhB in the plasma and tissue homogenates and the Y-axis represented the fluorescence intensity.

#### 4.8.2. Method Validation

##### Precision Test

Three quality control (QC) samples of high (100 µg mL^−1^), medium (50 µg mL^−1^), and low (1 µg mL^−1^) concentrations were prepared by adding different concentrations of DPC1-RhB solution to blank plasma and tissue homogenates. Five samples of each concentration were prepared and evaluated three times a day for three days. The QC sample concentrations and the intra- and inter-day precision were calculated using the standard curve.

##### Stability Test

The precision solution preparation method was used to prepare three concentrations of plasma and tissue homogenates with five replicates per group. The QC sample concentration was calculated using the standard curve, and the accuracy of the method was determined by comparing the results with the theoretical values.

##### Recovery Rates of Samples

The precision solution preparation method was used to prepare three concentrations of plasma and tissue homogenates with five replicates per group. The samples were stored at room temperature for 6 h, −4 °C for 24 h, and −20 °C for 7 d. The standard curve was used to calculate the recovery rate and stability of the samples at different storage conditions.

### 4.9. Pharmacokinetics and Tissue Distribution Studies

In pharmacokinetic studies, oral administration is the most common method of drug delivery. To mitigate the potential bias caused by a single dosing method and considering that certain drugs are better suited for non-oral administration, both oral and intraperitoneal routes were employed in this experimental design to investigate the tissue distribution of polysaccharides. Intraperitoneal injection, apart from being a simple procedure and causing minimal harm to mice, facilitates rapid absorption into the bloodstream, offering enhanced control over the drug’s absorption rate and concentration. In addition, different doses of administration may lead to different outcomes in vivo. To determine the absorption of DPC1, the pharmacokinetics of DPC1 were investigated at three different oral and intraperitoneal doses.

Pharmacokinetic experiments were conducted following references [36,44]. Combined with the results of the pre-tests, the high, medium, and low doses were established for oral and intraperitoneal administration. Briefly, healthy male BALB/c mice were randomly divided into six groups with 54 mice per group, and the sampling time points were set at 0.5, 1, 2, 3, 4, 6, 8, 12, and 24 h (*n* = 6). Mice housed in metabolic cages were fasted overnight before the experiment but were provided free access to drinking water. The mice in groups 1–3 received oral doses of 50, 100, or 150 mg kg^−1^ DPC1-RhB, respectively, whereas those in groups 4–6 received 25, 50, or 100 mg kg^−1^ DPC1-RhB, respectively, by intraperitoneal injection. Blood (0.5 mL) was collected from the retroorbital sinuses of the mice from each group into Eppendorf tubes containing anticoagulant following drug administration. After centrifuging the blood samples at 4 °C and 4000 rpm for 10 min, the plasma was collected and stored at −80 °C for later use. Plasma concentration-time curve (C-T) data were non-compartmentally analyzed using DAS (Drug and Statistics) 2.0. Pharmacokinetic parameters, including plasma clearance (CL), elimination half-life (T_1/2_), mean residence time (MRT), plasma concentration versus time curve (AUC), maximum peak concentration (C_max_), and F-corrected apparent volume of distribution, were calculated. All values are expressed as mean ± standard deviation.

After the administration of DPC1-RhB, the animals were euthanized at various time points (0.5, 1, 2, 3, 4, 6, 8, 12, and 24 h). Tissue from the liver, heart, spleen, lung, kidney, stomach, large intestine, and small intestine was harvested. Tissue homogenates were prepared by adding 9 volumes of PBS to each tissue sample, followed by homogenization using a multi-sample fast grinder, and centrifugation at 12,000 rpm for 10 min. The fluorescence intensities of the samples were measured using a multifunctional enzyme marker, and the drug concentration was calculated using the standard curve.

### 4.10. HE Toxicity Studies

HE section staining is a common method used to observe drug damage to tissue organs in mice [53]. Briefly, male BALB/c mice were randomly divided into four groups containing five mice per group, namely, the DPC1-RhB, RhB, DPC1, and blank groups. Before the experiment, all mice were fasted for 12 h but allowed free access to drinking water. DPC1-RhB was administered intraperitoneally at a dose of 150 mg kg^−1^, RhB was injected at a dose of 0.852 mg kg^−1^, and DPC1 was injected at a dose of 149.148 mg kg^−1^; an equal volume of saline was injected into the mice in the blank group. Mice were euthanized at a predefined time (24 h) following drug administration. Liver, heart, spleen, lung, kidney, stomach, large intestinal, and small intestinal tissues were removed, washed, and weighed. All tissues were fixed with 4% paraformaldehyde for 48 h, embedded in paraffin, cut into 5-µm-thick sections, and stained with hematoxylin–eosin (HE) [54]. The stained sections were then observed using a section scanner.

### 4.11. Statistical Analysis

All data are presented as the mean ± standard deviation (SD). SPSS 26.0 was used for statistical analysis. Differences between the groups were analyzed using one-way analysis of variance (ANOVA), with *p* < 0.05 being considered significant. Data that did not conform to the normal distribution were subjected to multiple comparisons between samples using the Kruskal–Wallis test. The figures were drawn with Origin 2019 software. The reaction equation was drawn with Chem Draw 19.0 software. All experiments were performed in triplicate.

## 5. Conclusions

In this study, the pharmacokinetics of the homogeneous polysaccharide DPC1 extracted from *P. cyrtonema* were evaluated for the first time. The concentration of DPC1 in biological samples was measured using fluorescent labeling. The success of the fluorescent labeling reaction was confirmed by UV, IR, ^1^H NMR, and GPC analyses. The fluorescence substitution was 0.568%. In vivo imaging results revealed that the fluorescence signal of DPC1-RhB started diffusing into the surrounding tissues at 0.5 h after administration and was basically metabolized completely at 24 h. Pharmacokinetic characteristics showed that oral administration and intraperitoneal administration were consistent with the features of a two-compartment model. DPC1-RhB has a short T_1/2_ and MRT_(0→∞)_ after administration and was rapidly absorbed into the bloodstream. Both C_max_ and AUC_(0→∞)_ demonstrated a positive correlation with the dose within each dose group. Drug concentrations in plasma showed an increasing and then decreasing trend over a 6 h period. In both delivery routes, DPC1-RhB was primarily distributed in the tissues of the heart, spleen, and lung, indicating that the drug has a targeted effect on these tissues. These findings provide a method for the study of similar polysaccharides and establish the basis for follow-up clinical research on DPC1.

## Figures and Tables

**Table 1:** Results of molecular weight determination of DPC1 and DPC1-RhB.

Sample		Mw (Da)	Mw/Mn	Mw Average	Mw/Mn Average	S	RSD
DPC1	1	3217	1.2230	3185	1.23	58.02	1.82%
	2	3280	1.2216				
	3	3161	1.2185				
	4	3127	1.2332				
	5	3191	1.2246				
	6	3132	1.2343				
DPC1-RhB	1	3097	1.2429	3134	1.24	27.14	0.87%
	2	3124	1.2371				
	3	3137	1.226				
	4	3125	1.2447				
	5	3144	1.2354				
	6	3179	1.2242				

**Table 2:** Standard curves of DPC1-RhB in plasma and tissues.

Sample	Linear Equation	R^2^
Plasma	*y* = 0.0292*x* − 0.4713	0.9986
Liver	*y* = 0.0477*x* + 0.1519	0.9994
Heart	*y* = 0.0627*x* − 0.2516	0.9989
Spleen	*y* = 0.0315*x* − 0.0675	0.9997
Lung	*y* = 0.0225*x* − 0.0446	0.9975
Kidney	*y* = 0.0397*x* + 0.2638	0.9932
Stomach	*y* = 0.0391*x* + 0.2153	0.9992
Large intestine	*y* = 0.0294*x* − 0.2787	0.9998
Small intestine	*y* = 0.0163*x* − 0.2572	0.9978

**Table 3:** Precision and accuracy of DPC1-RhB assay in mice plasma and tissues (*n* = 5).

Sample	Concentration (µg mL^−1^)	Measured Concentration (µg mL^−1^)	Accuracy (RE%)	Intra-Day Precision (RSD%)	Inter-Day Precision (RSD%)
Plasma	100	95.120 ± 10.037	−4.88	4.21	4.09
	50	54.360 ± 3.715	8.72	5.39	3.52
	1	1.045 ± 0.190	4.45	9.04	6.33
Liver	100	104.547 ± 8.171	4.55	5.21	6.34
	50	50.853 ± 6.037	1.71	3.84	2.11
	1	1.159 ± 0.222	15.93	9.73	7.18
Heart	100	97.008 ± 4.988	−2.99	10.56	8.38
	50	52.861 ± 6.728	5.72	7.42	4.33
	1	1.113 ± 0.139	11.32	4.26	6.17
Spleen	100	103.908 ± 14.948	3.91	8.29	6.78
	50	47.737 ± 3.439	−4.53	7.37	4.52
	1	0.905 ± 0.147	−9.52	10.42	4.39
Lung	100	105.076 ± 11.102	5.08	2.62	1.17
	50	49.049 ± 9.059	−1.90	8.36	11.28
	1	0.978 ± 0.195	−2.22	13.99	17.52
Kidney	100	95.718 ± 5.601	−4.28	7.49	3.77
	50	55.552 ± 6.178	11.10	2.59	6.61
	1	0.907 ± 0.213	−9.32	8.90	4.85
Stomach	100	93.824 ± 5.191	−6.18	4.43	2.21
	50	53.558 ± 5.068	7.12	2.62	4.94
	1	0.913 ± 0.306	−8.70	15.84	9.23
Large intestine	100	98.010 ± 9.509	−1.99	3.64	7.80
	50	47.670 ± 3.013	−4.66	1.72	4.63
	1	1.051 ± 0.179	5.10	2.06	10.37
Small intestine	100	103.840 ± 6.209	3.84	2.07	8.58
	50	49.865 ± 6.086	−0.27	7.70	2.91
	1	0.933 ± 0.371	−6.75	6.36	11.84

**Table 4:** Stability of DPC1-RhB in different matrices and different conditions (*n* = 5).

Sample	Concentration (µg mL^−1^)	Room Temperature for 6 h		−4 °C for 24 h		−20 °C for 7 d	
		Accuracy (%)	RSD (%)	Accuracy (%)	RSD (%)	Accuracy (%)	RSD (%)
Plasma	100	96.53	8.60	97.11	8.42	99.27	7.61
	50	101.25	9.22	98.98	7.29	102.20	4.99
	1	113.36	17.01	96.92	16.57	106.51	10.01
Liver	100	97.34	11.66	107.80	14.11	97.97	9.24
	50	108.80	8.53	112.16	5.86	98.82	17.75
	1	100.84	18.48	113.42	9.95	94.13	15.14
Heart	100	98.16	7.65	101.56	12.69	103.10	8.92
	50	102.55	9.09	96.87	13.42	101.87	3.43
	1	94.42	16.79	98.25	14.29	97.93	15.72
Spleen	100	101.03	18.28	90.51	8.48	104.63	9.94
	50	102.71	13.03	95.94	7.57	113.37	11.25
	1	98.73	18.56	102.54	13.91	93.02	12.40
Lung	100	105.72	9.72	88.11	7.53	100.98	8.57
	50	104.36	10.40	95.31	9.51	94.79	8.44
	1	87.11	18.04	99.56	11.56	96.00	15.35
Kidney	100	101.00	12.95	98.84	5.70	100.63	6.09
	50	110.61	13.99	107.19	16.95	99.76	14.87
	1	87.66	15.01	91.69	8.77	88.16	9.98
Stomach	100	96.83	10.73	99.39	15.44	94.01	8.79
	50	94.62	8.80	109.56	6.50	102.89	10.61
	1	106.14	17.24	97.44	15.37	95.91	11.53
Large intestine	100	95.02	7.62	100.60	9.73	97.23	5.38
	50	100.96	10.28	96.48	8.07	101.03	9.91
	1	98.30	16.12	105.78	13.94	98.98	11.55
Small intestine	100	95.75	4.85	91.39	12.10	98.76	8.25
	50	107.83	10.31	103.71	12.26	96.79	6.43
	1	107.98	17.33	96.93	15.24	99.39	9.76

**Table 5:** Pharmacokinetic parameters of DPC1-RhB after oral administration at three different doses (ig: 50 mg kg^−1^, 100 mg kg^−1^, and 150 mg kg^−1^) (*n* = 6).

Parameter	Dose		
	50 mg kg^−1^	100 mg kg^−1^	150 mg kg^−1^
T1/2 (h)	2.517 ± 0.867	3.091 ± 1.468	3.419 ± 1.853
AUC(0→∞) (mg h L−1)	104.411 ± 21.070	147.986 ± 16.240 *	245.938 ± 44.124 **^#^
CLZ/F (L h−1 kg−1)	0.495 ± 0.094	0.683 ± 0.076 **	0.625 ± 0.097 *
MRT(0→∞) (h)	5.129 ± 1.972	4.664 ± 1.607	3.494 ± 1.037
C_max_ (mg L−1)	34.697 ± 9.425	70.236 ± 10.519 **	117.374 ± 13.582 **^,#^
Tmax (h)	0.917 ± 0.204	1.000 ± 0.000	1.000 ± 0.000
Vz/F (L kg−1)	1.823 ± 0.821	3.079 ± 1.651	2.897 ± 1.152

* *p* < 0.05, ** *p* < 0.01 compared to the 50 mg kg^−1^ group; ^#^ *p* < 0.01 compared to the 100 mg kg^−1^ group.

**Table 6:** Pharmacokinetic parameters of DPC1-RhB after intraperitoneal administration at three different doses (ip: 25 mg kg^−1^, 50 mg kg^−1^, and 100 mg kg^−1^) (*n* = 6).

Parameter	Dose		
	25 mg kg^−1^	50 mg kg^−1^	100 mg kg^−1^
T1/2 (h)	0.478 ± 0.211	0.950 ± 0.400	1.826 ± 0.823
AUC(0→∞) (mg h L−1)	93.869 ± 11.636	180.113 ± 35.384 **	332.926 ± 27.341 **^,#^
CLZ/F (L h−1 kg−1)	0.270 ± 0.037	0.286 ± 0.051	0.302 ± 0.025
MRT(0→∞) (h)	0.855 ± 0.112	1.514 ± 0.378	1.613 ± 0.493
C_max_ (mg L−1)	74.944 ± 14.915	110.921 ± 11.453 **	212.231 ± 12.600 **^,#^
Tmax (h)	0.330 ± 0.186	0.330 ± 0.186	0.273 ± 0.176
Vz/F (L kg−1)	0.183 ± 0.068	0.370 ± 0.110	0.795 ± 0.374

** *p* < 0.01 compared to the 25 mg kg^−1^ group; ^#^ *p* < 0.01 compared to the 50 mg kg^−1^ group.

## Data Availability

Data will be made available on request.

## Supplementary Materials

The following supporting information can be downloaded at: https://www.mdpi.com/article/10.3390/ph17030343/s1, Table S1. In vivo distribution of DPC1-RhB in mice after oral administration at three different doses (*n* = 6). Table S2. In vivo distribution of DPC1-RhB after intraperitoneal administration at three different doses (*n* = 6).
