# Supplementary material for: In Vivo Pharmacokinetic Study of Polygonatum cyrtonema Polysaccharide DPC1 after Oral and Intraperitoneal Administration
Source: Pharmaceuticals (Basel). 2024 Mar 6;17(3):343. doi: 10.3390/ph17030343 (PMC10974776; doi:10.3390/ph17030343)
Supplement: Supplementary file 1 [file pharmaceuticals-17-00343-s001.zip › pharmaceuticals-2898482-supplementary.pdf]

Table S1. *In vivo* distribution of DPC1-RhB in mice after oral administration at three different doses (n = 6).

Sample	Dose (mgkg-1)	30min	1h	2h	3h	4h	6h	8h	12h	24h
Plasma	150	51.469±4.764	117.373±13.580*	44.174±6.782&	21.618±5.042*&#	9.752±6.105*&#	10.865±7.492*&#	2.722±4.004*&#★	0.758±3.095*&#★	-
	100	32.991±3.792	70.236±10.519*	14.076±6.632*&	9.068±2.460*&	2.514±6.835*&	13.822±4.028*&	-	-	0.657±1.873*&★α
	50	16.531±3.254	33.952±10.980	14.764±5.689	6.462±4.579*&	9.486±3.214&	-	3.825±7.671*&#	0.034±1.494*&#§	1.233±1.444*&#
Liver	150	5.312±1.834	1.310±0.620*	4.009±1.181&	3.829±2.462&	2.182±1.470*	4.855±1.627&§	4.257±2.315&§	2.533±1.558*α	3.669±1.409&
	100	4.685±2.201	2.524±1.442	2.918±0.853	3.424±1.712	4.678±1.816	2.298±1.668	3.890±1.417	2.483±1.646	4.414±2.474
	50	0.975±0.847	0.856±0.845	2.065±1.472	3.125±1.420	-	3.961±2.619	2.218±1.578	2.929±1.838	1.086±0.880
Heart	150	20.263±2.349	25.327±3.563	26.117±2.900	28.236±3.093	27.575±3.448	27.215±2.277	27.650±3.918	22.327±4.374	12.516±3.794★§αβ
	100	14.071±1.971	20.782±3.058	22.300±3.298	21.759±2.250	23.692±3.611	25.004±3.838*	22.571±3.376	18.883±5.492	9.726±1.862§αβ
	50	7.446±0.801	13.247±2.985*	12.658±2.610*	13.111±1.796*	15.401±2.400*	17.456±1.577*&#★	18.529±4.075*&#★§	12.704±3.141*αβ	8.366±1.508&#★§αβθ
Spleen	150	15.478±2.077	15.896±2.282	17.827±3.349	19.801±2.426	27.058±4.537	22.328±3.209	14.356±1.572§	14.501±1.198§	8.949±1.445★§α
	100	11.275±2.453	11.882±1.936	14.439±3.174	17.178±2.397	19.827±1.805*&	17.083±2.181	11.485±1.600§	12.513±1.253	6.584±0.933★§α
	50	9.549±1.932	11.353±2.245	11.587±1.162*	12.113±1.652*	15.601±1.960*&#★	14.333±1.587*&#★	10.282±1.335§α	9.131±1.285&#★§α	4.873±1.064*&#★§αβθ

Lung	150	16.365±2.167	20.602±2.060*	23.032±1.648*&	24.674±1.740*&	21.385±1.282*★	18.716±2.342*#★§	15.891±1.698&#★§α	16.313±2.114&#★§α	2.108±0.803*&#★§αβθ
	100	12.387±1.752	16.256±1.614*	20.580±2.335*&	19.486±1.247*&	17.607±1.590*#	15.402±1.138*#★§	14.046±3.375&#★§	8.802±1.618*&#★§αβ	2.888±0.990*&#★§αβθ
	50	9.288±1.322	12.582±1.843	18.562±2.232*	13.106±0.752	9.422±1.281#	10.028±2.375	11.693±0.612	6.901±1.526#★	2.004±0.899&#★β
Kidney	150	3.712±0.810	4.737±0.688	3.989±1.287	8.703±2.077	9.653±2.261*	8.010±2.775	4.216±1.056	3.658±0.882§	3.082±1.633§
	100	1.544±0.548	3.169±1.125	2.506±0.949	4.072±1.470	4.031±1.429	7.510±1.725*#	4.246±0.570*	2.630±0.744α	-
	50	-	0.520±0.385	1.951±0.880	4.226±0.848&	4.867±1.226&	7.314±1.228&#	1.474±0.308α	-	-
Stomach	150	4.052±2.271	6.657±1.931	46.560±8.091*&	84.262±5.418*&	46.869±7.705*&★	3.481±2.281#★§	1.055±0.981&#★§	-	-
	100	3.124±1.695	5.697±1.644	12.394±3.655*	68.935±6.460*&#	12.636±5.802★	1.373±0.820&#★	-	-	-
	50	1.388±0.956	4.008±1.641	8.361±3.669	20.005±4.956*&#	5.427±2.428★	1.705±1.193★	0.298±0.443&★	-	-
Large intestine	150	17.846±2.282	26.644±4.248*	27.110±4.191*	38.963±6.805*&#	88.416±7.379*&#★	70.180±5.113*&#★§	31.470±3.976*★§α	14.178±2.097&#★§αβ	10.028±2.368*&#★§αβ
	100	14.549±2.332	20.517±2.593	25.587±1.582*	36.106±3.976*&#	75.614±8.679*&#★	64.667±4.678*&#★	17.763±1.340#★§α	11.481±3.776&#★§α	9.783±2.334&#★§αβ
	50	21.978±4.112	21.812±4.355	22.719±4.746	28.199±3.305	54.844±6.373	45.272±5.192	13.774±2.592§α§	13.047±2.615§α	9.981±3.372★§α
Small intestine	150	171.719±15.949	255.659±23.957	167.378±15.249	41.691±11.215	32.334±5.572	5.799±3.187*&#	9.070±3.177*&	10.428±4.218*&	10.628±3.360&
	100	151.974±10.091	200.593±22.715	140.669±17.214&	25.482±7.236*&#	17.677±5.915*&#	5.768±2.754*&#★	15.890±2.820*&#α	12.610±4.655*&#	7.536±3.681*&#★

50	103.437±11.542	157.882±13.345	61.588±10.247	18.932±3.950	12.447±4.117$^{\&}$	12.121±3.493$^{\&}$	10.947±3.422$^{*\&}$	12.046±2.887$^{\&}$	9.223±5.366$^{*\&}$

$^{*}P < 0.05$, versus the 0.5 h group; $^{\&}P < 0.05$, versus the 1 h group; $^{\#}P < 0.05$, versus the 2 h group; $^{\star}P < 0.05$, versus the 3 h group; $^{\S}P < 0.05$, versus the 4 h group；$^{\alpha}P < 0.05$, versus the 6 h group; $^{\beta}P < 0.05$, versus the 8 h group；$^{\theta}P < 0.05$, versus the 12 h group.

Table S2. *In vivo* distribution of DPC1-RhB after intraperitoneal administration at three different doses (n = 6).

Sample	Dose ($mgkg^{-1}$)	10min	30min	1h	2h	3h	4h	6h	8h	12h	24h
Plasma	100	205.118±10.858	184.683±31.954	125.598±15.432$^{\partial}$	50.280±3.344$^{\partial *\&}$	33.500±14.553$^{\partial *\&}$	3.592±3.073$^{\partial *\&\#}$	-	1.803±3.969$^{\partial *\&\#}$	-	-
	50	106.136±13.268	102.750±15.122	82.493±8.253	12.682±6.978$^{\partial *}$	14.882±11.133$^{\partial *}$	-	-	-	0.465±6.634$^{\partial *\&}$	-
	25	72.395±17.064	65.566±7.443	48.096±9.471*	7.343±6.019$^{\partial *\&}$	-	-	-	-	-	-
Liver	100	4.230±2.135	3.092±0.906	6.498±2.970*	4.751±1.666	3.225±2.496$^{\&}$	2.635±1.511$^{\&}$	3.355±1.938$^{\&}$	4.395±2.152$^{\&}$	1.963±0.867$^{\&\#\beta}$	-
	50	2.124±1.555	3.182±1.631	2.903±1.317	4.200±1.714	3.051±1.634	2.118±1.992	2.825±1.587	2.518±0.642	-	1.670±1.353
	25	-	1.736±0.859	2.416±1.379	4.263±2.425	-	4.740±2.962	-	3.355±1.849	1.013±0.819	-
Heart	100	9.379±2.968	16.237±2.587	23.827±3.801$^{\partial}$	25.247±2.966$^{\partial *}$	22.286±7.587	19.454±7.239	15.040±3.994$^{\#}$	13.014±1.948$^{\&\#}$	9.010±2.194$^{*\&\#}$	5.228±0.562$^{*\&\#\beta}$
	50	5.333±1.428	13.374±2.026$^{\partial}$	19.113±1.421$^{\partial *}$	21.047±2.831$^{\partial *}$	20.086±4.401$^{\partial *}$	16.758±4.449$^{\partial \#}$	12.016±4.291$^{\partial \&\#\star\S}$	10.513±2.870$^{\partial \&\#\star\S}$	7.281±1.650$^{*\&\#\star\S\alpha}$	5.097±1.365$^{*\&\#\star\S\alpha\beta}$
	25	3.254±0.583	11.979±2.776$^{\partial}$	15.645±3.478$^{\partial}$	17.719±3.036$^{\partial}$	16.492±4.947	12.623±6.071	9.024±3.888	7.166±2.690$^{\&\#}$	5.240±1.641$^{*\&\#}$	3.680±1.211$^{*\&\#\star}$
Spleen	100	21.356±2.562	31.016±2.827$^{\partial}$	32.481±3.320$^{\partial}$	27.136±2.751$^{\partial *\&}$	26.510±3.521$^{\partial *\&}$	28.145±2.922$^{\partial \&}$	23.123±2.169$^{*\&\#\star\S}$	25.141±2.851$^{\partial *\&}$	22.333±1.989$^{*\&\#\star\S}$	14.829±2.267$^{\partial *\&\#\star\S\alpha\beta\theta}$
	50	15.513±1.703	23.287±2.824	25.598±1.997$^{\partial}$	22.582±2.851	25.129±2.731$^{\partial}$	22.996±3.200	19.376±1.903	20.004±2.214	18.667±2.186	11.942±2.352$^{*\&\#\star\S}$
	25	11.767±1.654	17.196±1.302$^{\partial}$	18.533±2.065$^{\partial}$	17.016±3.936$^{\partial}$	18.169±2.644$^{\partial}$	16.772±3.051$^{\partial}$	15.434±3.241$^{\partial \&}$	15.810±2.086$^{\partial}$	14.746±2.564$^{\partial \&\star}$	7.781±1.879$^{\partial *\&\#\star\S\alpha\beta\theta}$

Lung	100	21.464±1.536	22.651±2.867	26.676±2.803∂	24.222±2.951	22.059±2.785	34.046±3.895∂ ★*#	28.748±2.516∂ ★	24.738±2.993§	11.802±2.457& #§αβ	0.787±0.339* &#★§αβ
	50	16.839±3.067	18.595±3.371	20.130±1.651	18.701±2.124	19.093±2.324	26.854±3.974∂ *&#★	24.116±4.191∂ *&#★	22.111±2.834∂ *§	9.382±2.552∂* &#★§αβ	-
	25	10.587±1.979	12.367±1.202	17.908±1.469∂ *	14.943±2.103∂	13.525±2.029&	20.582±3.247∂ *★	16.061±1.984∂ *	14.056±2.373& §	4.279±1.471&# ★§αβ	-
Kidney	100	3.950±0.700	14.033±1.020∂	14.264±1.433∂	13.184±0.777∂	15.286±1.257∂	14.117±2.400∂	8.155±0.942* &★	6.555±0.809* &#★§	7.425±0.595* &★§	-
	50	3.453±0.899	10.234±2.236∂	10.892±2.386∂	11.682±1.402∂	10.962±1.382∂	9.753±0.885∂#	6.610±0.650∂* &#★§	4.181±0.948* &#★§α	5.865±0.866∂* &#★§β	-
	25	-	4.131±0.893	4.451±0.952	7.838±1.303*&	8.258±0.646*&	4.542±0.785#★	2.605±0.892* &#★§	4.041±0.799#★ α	1.773±0.610* &#★§β	-
Stomach	100	3.682±1.663	6.044±1.186∂	6.925±1.653∂	17.292±2.333∂ *&	5.309±1.259#	4.578±1.827&#	-	1.799±0.876∂* &#★§	0.584±1.074∂* &#★§	-
	50	3.318±1.877	5.818±1.518	5.424±0.713	15.807±1.887∂	4.052±1.791#	2.779±0.957#	-	1.063±0.540* &#★	1.160±0.892* &#★	-
	25	-	4.808±1.581	6.096±1.669	10.340±2.084* &	5.434±1.849#	-	-	-	-	-
Large intestine	100	27.185±1.483	31.074±1.471	37.989±1.390∂	24.866±3.220* &	29.672±4.873	27.089±1.001&	21.978±5.195* &★	21.238±2.053* &★	19.385±5.219∂ *&★	10.908±0.964∂ *&#★§
	50	20.291±3.534	23.578±3.185	32.717±2.334	21.168±4.003	21.370±2.828	16.074±3.125&	22.008±3.812	21.147±3.528	16.581±4.406&	10.144±3.118* &
	25	13.908±2.867	21.629±1.232∂	28.642±1.809∂	18.849±3.373∂ &	17.161±0.766&	18.925±3.485∂ &	17.442±4.216&	17.819±2.266&	15.887±2.788* &	11.696±2.898* &#★§αβ
Small intestine	100	20.578±5.854	55.242±6.053	22.665±4.000	34.387±5.979	19.598±4.936	15.562±2.992*	23.134±4.727	15.505±4.359*	13.994±3.522* #	9.348±3.686*#
	50	19.557±4.023	40.676±4.630	17.585±3.542	31.309±4.928	24.708±5.194	20.100±4.099	18.230±4.006	15.328±3.072* #	14.537±3.995* #	11.839±2.653* #★

25	16.713±6.969	34.133±5.603∂	21.953±1.286*	37.649±5.225∂	21.875±5.088*#	20.531±5.327*#	15.912±4.517*&#★	13.339±4.453*&#★§	11.042±4.296∂*&#★§	9.491±2.905∂*&#★§α

∂$P < 0.05$, versus the 0.17 h group; *$P < 0.05$, versus the 0.5 h group; &$P < 0.05$, versus the 1 h group; #$P < 0.05$, versus the 2 h group; ★$P < 0.05$, versus the 3 h group; §$P < 0.05$, versus the 4 h group；α$P < 0.05$, versus the 6 h group; β$P < 0.05$, versus the 8 h group；θ$P < 0.05$, versus the 12 h group.
